# Investigating Population Structure of Sea Lamprey (*Petromyzon marinus*, L.) in Western Iberian Peninsula Using Morphological Characters and Heart Fatty Acid Signature Analyses

**DOI:** 10.1371/journal.pone.0108110

**Published:** 2014-09-26

**Authors:** Maria João Lança, Maria Machado, Catarina S. Mateus, Marta Lourenço, Ana F. Ferreira, Bernardo R. Quintella, Pedro R. Almeida

**Affiliations:** 1 Escola de Ciências e Tecnologia, Departamento de Zootecnia, Universidade de Évora, Évora, Portugal; 2 Instituto de Ciências Agrárias e Ambientais Mediterrânicas, Universidade de Évora, Évora, Portugal; 3 Centro de Oceanografia, Faculdade de Ciências, Universidade de Lisboa, Lisboa, Portugal; 4 Museu Nacional de História Natural e da Ciência & Centro de Biologia Ambiental, Universidade de Lisboa, Lisboa, Portugal; 5 Departamento de Biologia Animal, Faculdade de Ciências, Universidade de Lisboa, Lisboa, Portugal; 6 Escola de Ciências e Tecnologia, Departamento de Biologia, Universidade de Évora, Évora, Portugal; Scottish Association for Marine Science, United Kingdom

## Abstract

This study hypothesizes the existence of three groups of sea lamprey *Petromyzon marinus* L. in Portugal (North/Central group, Tagus group, and Guadiana group), possibly promoted by seabed topography isolation during the oceanic phase of the life cycle. Within this context, our purpose was to analyze the existence of a stock structure on sea lamprey populations sampled in the major Portuguese river basins using both morphological characters and heart tissue fatty acid signature. In both cases, the multiple discriminant analysis revealed statistically significant differences among groups, and the overall corrected classification rate estimated from cross-validation procedure was particularly high for the cardiac muscle fatty acid profiles (i.e. 83.8%). Morphometric characters were much more useful than meristic ones to discriminate stocks, and the most important variables for group differentiation were eye length, second dorsal fin length and branchial length. Fatty acid analysis showed that all lampreys from the southern Guadiana group were correctly classified and not mixing with individuals from any other group, reflecting a typical heart fatty acid signature. Our results revealed that 89.5% and 72.2% of the individuals from the Tagus and North/Central groups, respectively, were also correctly classified, despite some degree of overlap between individuals from these groups. The fatty acids that contributed to the observed segregation were C16:0; C17:0; C18:1ω9; C20:3ω6 and C22:2ω6. Detected differences are probably related with environmental variables to which lampreys may have been exposed, which leaded to different patterns of gene expression. These results suggest the existence of three different sea lamprey stocks in Portugal, with implication in terms of management and conservation.

## Introduction

European populations of sea lamprey (*Petromyzon marinus* L.) have declined over the last 30 years [1], [2], and several authors have pointed out a reduction in sea lamprey abundance in Portuguese rivers [3], [4]. Sea lampreys can be found in all major Portuguese river basins, being more abundant in the central and northern regions of the country [3]. Due to the reduction in population abundance and the anthropogenic pressures to which this species is subjected, in Portugal it is classified as “Vulnerable” in the Red List of Threatened Vertebrates [4].

Whereas the continental phase of lampreys' life cycle is well known, the oceanic phase remains a mystery, with available data resuming to a few accidental captures of host species with scars or, occasionally, lampreys still attached to the fish or cetaceans [5]. A limited record of 80 sea lampreys captured in the northwest Atlantic indicated that almost all individuals with less than 39 cm long where taken in bottom trawls on the continental shelf or in coastal trap nets, whereas most animals with more than 56 cm long were captured in mid-water trawls along the shelf edge or over the continental slope [6]. Evidence that sea lamprey might not show homing behaviour first emerged following a tagging study with a landlocked population of the Great Lakes [7], and was then corroborated using genetic analysis on anadromous populations captured along the east coast of North America [8], [9].

The anatomy and physiology of an individual is sensitive both to genetic and environmental factors, which are responsible for phenotypic variation reflecting morphological characteristics [10]. In meristic terms, the effect of abiotic factors during ontogeny may result in significant differences between individuals of the same population, [11]. Morphometric characters are exposed to the same abiotic factors for an even longer period of time, which may increase the susceptibility of having more differences [12]. If those differences are ecologically significant and constant in time, they may allow the identification of individuals of different populations or stocks [13]. Morphometric variables measured in the cephalic region of sea lamprey larvae were found to be more suitable for a morphological analysis of geographic variation between Portuguese river basins [14]. Meristic characters were also assessed but the discriminatory power between groups, i.e. river basins, was comparatively weaker.

The concept of stock is fundamental for both fisheries and endangered species management [15]. A stock can be defined as a population or portion of a population of which all members are characterized by similarities which are not heritable, but are induced by the environment, and which include members of several different subpopulations [16]. Unit stocks can also be defined as characteristic populations or sets of subpopulations within subareas of the geographic range of a species [17], or as “… an intraspecific group of randomly mating individuals with temporal and spatial integrity” [13].

Spawning areas are normally clearly distinguished among the different stocks, but since fish may undertake considerable migrations, catches may also consist of fish from several stocks. For this reason, much work has been carried out to find characters that can be used for stock identification [18]. Waldman *et al.* [19] suggested that stock identification could be based upon catch data, tag recoveries, meristics, morphometrics, scale morphology, parasites, and cytogenetic: protein electrophoresis, monogenetic, mitochondrial DNA and nuclear DNA.

One of the limitations when using fatty acid profiles of a tissue as biomarkers and/or to characterize species, subspecies, populations, or stocks, is that the fatty acid profile under analysis can be influenced by various environmental factors, including the diet [20]. However, when fatty acid profiles are used for identification, the assumption is that the composition of fatty acids in membrane phospholipids is genetically controlled and stable over time, and therefore the phospholipid fatty acids may be used as a natural marker over a longer timescale [21]. Several studies have indicated genetic control of the fatty acid composition in the heart lipids although the impact of environmental factors could not be excluded [18], [21]. The lipid composition of cardiac skeletal muscle has a high level of polar lipids incorporated in the membrane phospholipid pool, so its fatty acyl structure restricts the ability of the acyl chains to reflect diet [22], and because of the specialized functions of these lipids on membranes, this lipid class is relatively robust to dietary changes. For the reasons explained above, fatty acids of cardiac skeletal muscle may serve as natural markers for the identification of stocks [21], [23]. In the last decade, several reports have suggested that fatty acid composition of phospholipids in some body tissues (e.g. heart tissue, brain, eggs) have a stable genetics basis, making these tissues appropriate for stock identification [18], [20], [24], [25]. Many fish species such as herring (*Clupea harengus* L.), striped bass (*Morone saxatilis* Walbaum, 1792) and cod (*Gadus morhua* L.) had been studied with this approach looking for possible stock differences [18], [26], [27].

**Figure 1:**
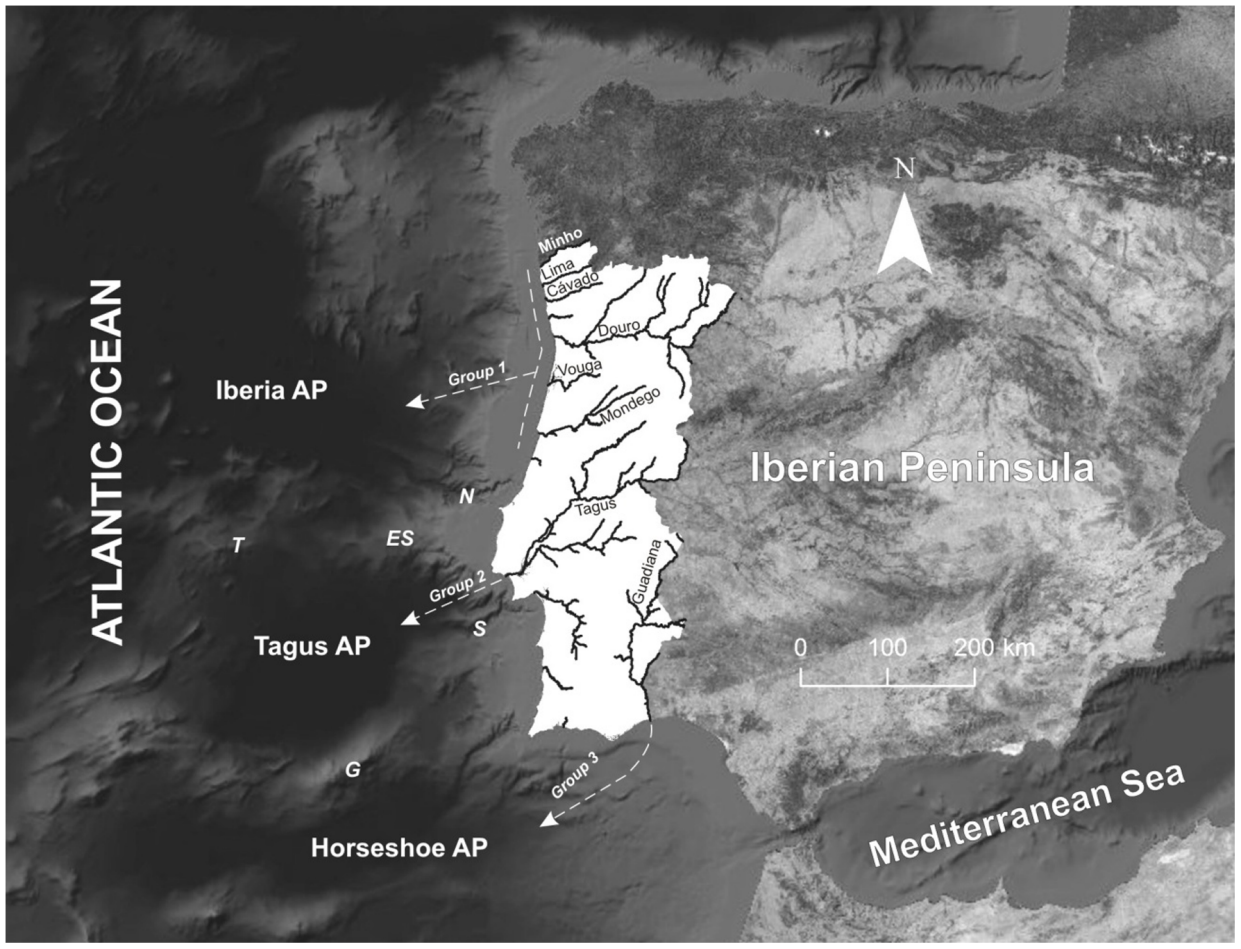
Location of the river basins from which sea lamprey individuals were collected. Formation of the three groups (testing hypothesis) based on the geographical location of the river mouth and the proximity to western Iberian oceanic areas with the representation of the seamounts and canyons that contour the three abyssal plains. Acronyms: Iberia AP - Iberia Abyssal Plain; Tagus AP – Tagus Abyssal Plain; Horsheshoe AP – Horsheshoe Abyssal Plain; T – Tore Seamount; ES – Estremadura Spur; G – Gorringe Bank.

Within this context, we hypothesize the existence of three sea lamprey groups in Portugal, possibly promoted by the seabed topography isolation during the oceanic phase of the life cycle; three large abyssal plains, and adjacent continental slopes, occur off western Iberian Peninsula: the Iberia Abyssal Plain in the north, the Tagus Abyssal Plain in the centre and the Horseshoe Abyssal Plain in the south. The Iberia Abyssal Plain is separated from the Tagus Abyssal Plain by the Estremadura Spur and the Tore Seamount, and by the Nazaré Canyon (continental shelf). The Tagus Abyssal Plain is separated from the Horseshoe Abyssal Plain through the Gorringe Bank and the Setúbal Canyon (Fig. 1).The hypothesis presented in this study is associated with the assumption that the bulk of the parasitic attacks are directed towards benthic hosts, which are believed to have restricted dispersal capability when compared with the more mobile pelagic species, and thus exchanges between lamprey feeding areas (i.e. groups) are strongly reduced. This hypotheses is supported by two evidences: (i) recent discoveries indicate a shorter hematophagous feeding stage (∼1 year) [28], first reported to last from 23 to 28 months [29]; and (ii) there are no capturing records of adult lampreys or fish with wounds, compatible with potential lamprey attachments, in the data collected from annual surveys performed in the Portuguese continental shelf by IPMA I. P., the Portuguese fisheries laboratory (Yorgos Stratoudakis, pers. comm.). The short adult feeding stage attributed to the sea lamprey reduces considerably their dispersion capability in the marine environment, and this fact, together with the absence of evidences of adult lampreys feeding in the continental shelf, support the present study hypotheses that postulates that the majority of the growth during the parasitic feeding stage are related with attachments to benthic hosts species that live in the continental slope and/or abyssal plains. To test this hypothesis we analysed (i) morphological differentiation, and (ii) heart tissue phospholipid fatty acid profile between sea lamprey adults from the main Portuguese river basins, divided in the three groups mentioned above. In parallel, we performed analysis of genetic differentiation among the exact same groups using 12 microsatellite loci (results not shown, unpublished data), but no differences were found among the three groups at this level. To end, we discuss on the possibility of the existence of three sea lamprey stocks off western Iberian Peninsula, distinct by segregation in the trophic phase of the life cycle, and make some considerations and recommendations for conservation.

## Methods

### Ethics statement

Sampled sea lampreys were transported alive to the laboratory in a 0.4 m^3^ capacity tank equipped with proper life support system including aeration, external filter and temperature control. In the laboratory, the individuals were first immersed in cold water to minimize handling stress and pain sensibility and sacrificed through decapitation method. This study was carried out in strict accordance with the recommendations present in the Guide for the Care and Use of Laboratory Animals of the European Union – in Portugal represented by the Decree-Law n°129/92, Portaria n°1005/92. Approval by a named review board institution or ethics committee was not necessary as the final model for ethical experimentation using fish as biological models was not implemented in Portuguese research units at the time of experimentation. This work was conducted under an institutional license for animal experimentation and a personal license to first author Maria João Lança and the co-authors Pedro R. Almeida and Bernardo R. Quintella, issued by the Direcção-Geral de Veterinária (DGV), Portuguese Ministry of Agriculture, Rural Development and Fisheries.

### Sampling

Sea lamprey spawners were captured in March 2008 during the peak of their spawning migration in eight river basins: Minho (41°52′N; 08°50′W), Lima (41°41′N; 08°49′W), Cávado (41°32′N; 08°47′W), Douro (41°08′N; 08°40′W), Vouga (40°39′N; 08°43′W), Mondego (40°8′N; 08°50′W), Tagus (39°03′N; 08°47′W) and Guadiana (37°38′N; 07°39′W). No specific permissions were required for sampling in these locations because the adult lampreys were captured by local fishermen in designated professional fishing areas. This study was conducted with a species considered “Vulnerable” by the Portuguese Red List of Threatened Vertebrates but general permits for field sampling fish (including *P. marinus*) were accredited by the Autoridade Florestal Nacional (AFN).

A total of 224 sea lampreys were collected, including about 30 individuals from each river basin. Specimens from each river basin were grouped *a priori* to test the hypothesis of stock fragmentation promoted by the seabed physiographic features during the oceanic parasitic phase. We defined three groups based on the geographical location of the river mouth, namely, the proximity to western Iberia oceanic areas (Fig. 1). Group 1 includes individuals captured in the Minho, Lima, Cávado, Douro, Vouga and Mondego river basins; Group 2 collects specimens captured in River Tagus; and Group 3 includes individuals from River Guadiana (Fig. 1).

Detailed temperature–salinity distribution in the Northeast Atlantic, the region encompassing the three large abyssal plains (i.e. Iberia, Tagus and Horseshoe), is available at the web-site of the Centre of Oceanography of the University of Lisbon (http://co.fc.ul.pt/en/data) [30].

### Morphological characters

**Figure 2:**
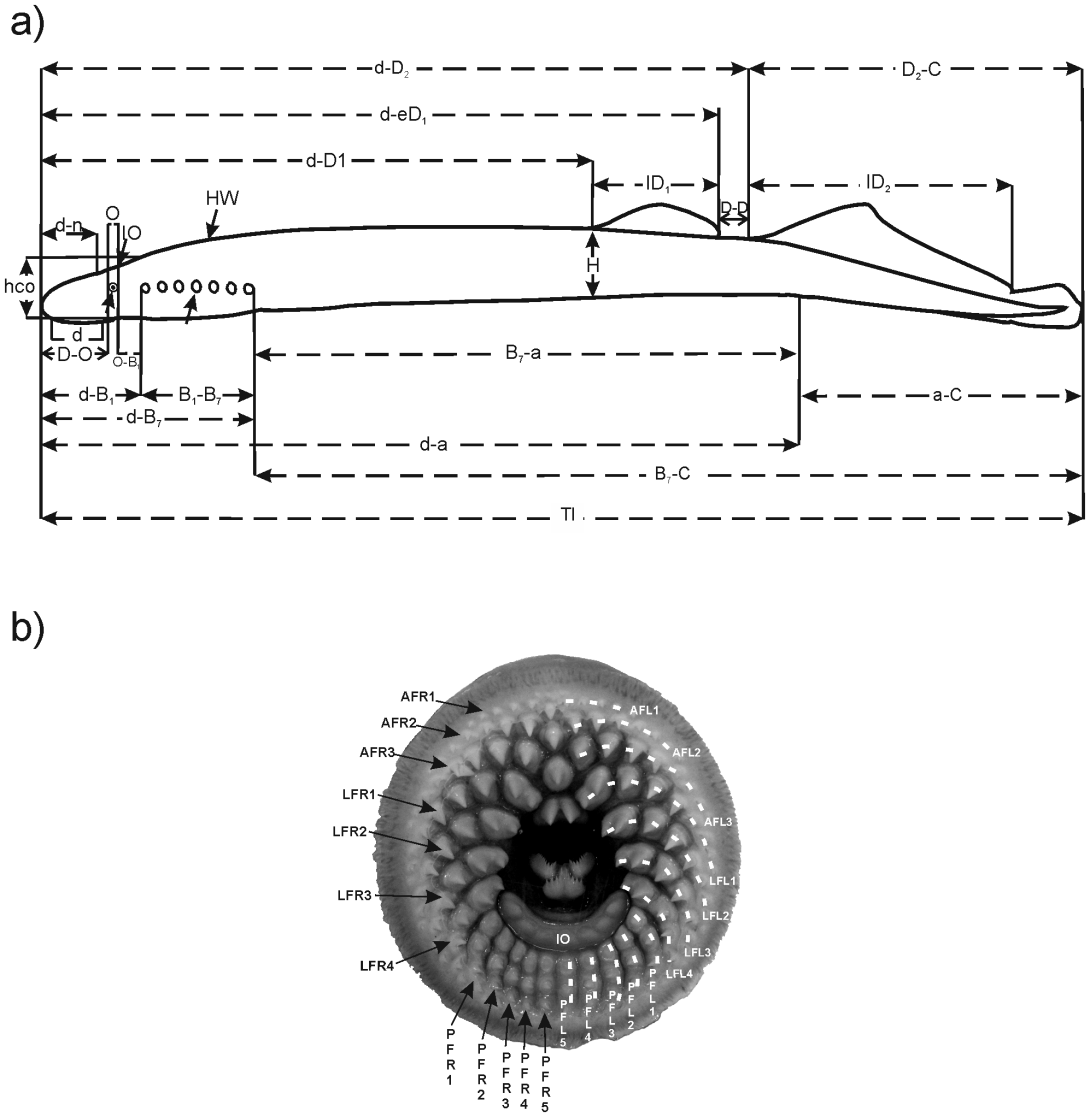
Schematic representation of the morphological features recorded for the analysis of geographic variation of sea lamprey in Portugal. (a) lateral view outline with the representation of the measured morphometric characters: TL, total length; d, disc length; d-a, distance between disc and anus; a-C, tail length; B_7_-C, postbranchial length; B_7_-a, trunk length; d-D_1_, predorsal distance; d-eD_1_, distance between disc and posterior end of first dorsal fin; d-D_2_, distance between disc and base of second dorsal fin; D_2_-C, dorsal part of caudal fin length; lD_1_, first dorsal fin length; lD_2_, second dorsal fin length; D-D, distance between dorsal fins; H, body depth; d-O, preocular distance; O, eye diameter; O-B_1_, postocular length; Hco, head depth; d-B_1_, prebranchial length; B_1_-B_7_, branchial length; d- B_7_, head length; d-n, prenostril length; IO, interocular distance; HW, head width; (b) photograph of the oral disc with the representation of the counted meristic characters: AF, anterior field; LF*_R_*, lateral right field; LF*_L_*, lateral left field; PF, posterior field; SO, supraoral lamina; L, lingual lamina; IO, infraoral lamina; TNteeth, total number of teeth; AFteeth, number of teeth in the anterior field; LFteeth, number of teeth in the lateral field; PFteeth, number of teeth in the posterior field; TNrows, total number of rows; AFrows, number of rows in the anterior field; LFrows, number of rows in the lateral field; PFrows, number of rows in the posterior field; IOcusps, number of cusps in the infra-oral lamina).

A total of 34 morphological characters were used: 24 morphometric and 10 meristic (Fig. 2), following [31]–[33].

All the 224 captured lampreys were used for the morphometric analysis. The morphometric characters were measured using graduated scales (±0.5 mm) and callipers (±0.5 mm; Fig. 2a). A sub sample of 201 lampreys was used for the meristic analysis. The oral disc of each individual was photographed (Kodak Z740) to count the meristic characters (Fig. 2b). To standardize the procedure, the picture was taken through an acrylic plate with the oral disc always opened to its maximum amplitude. A graphical scale was used to calibrate each image. The pictures were analyzed and processed using the Image J software [34] to count the number of teeth and rows in the anterior, laterals and posterior fields of the lamprey oral disc. The adopted teeth terminology (Fig. 2b) follows that proposed by Vladykov and Follett [31]. Trunk myomeres were counted between the anterior edge of the cloacal slit and the posterior edge of the last branchial opening, following [32]. All counts and measurements were made on the left side of the body.

Total mass (±0.01 g) of each individual was determined using a precision balance (Kern 440-36).

To test the hypothesis of sexual dimorphism in morphometric and meristic characters of *P. marinus*, the gender of all individuals was confirmed with histological slides, prepared with sections of reproductive organs according to the standard protocol of [35]. Histological slides were observed using a stereomicroscope (Leica DM 2000).

### Tissue preparation and collection

Data on body total mass (TM, nearest g) and total length (TL, nearest mm) was registered for each sea lamprey.

Data on heart total mass (HTM, nearest g) and gender was reported for each sea lamprey (see previous section for gender determination). Sex ratio (male/female), heart total mass/body gutted mass ratio (HTM/BGM, expressed in percentage) and heart percentage of water loss (H_water_) were also determined.

The heart was rapidly excised and rinsed in ice-cold 0.9% NaCl solution. Heart was then sliced and frozen between the tongues of an aluminum clamp that was cooled in liquid nitrogen. The frozen tissue heart samples were stored in aluminum canisters at −80°C until laboratorial processing.

### Heart tissue lipid extraction and fatty acid analysis

Pre-testing (random subsample of 8 individuals per river basin) determination of heart total lipids, neutral lipids and polar lipids revealed that more than 90 percent of the lipids present in heart tissue were polar lipids, resulting in all further analyses for fatty acid profile characterization were only done in polar lipid class. Lipid extraction was made according to the method described by Lança *et al*. [36]. Briefly, heart tissue total lipids were extracted using a Dionex 100 accelerated solvent extractor (ASE). To prepare for extraction, aliquots of 1 g portion of heart muscle samples were weighed on an analytical balance (Mettler AT201; Greifensee, Switzerland) and their masses were recorded to the nearest 0.01 mg. Tissue samples were then lyophilized until constant mass to determine the percentage of water loss and aliquots of heart tissue with 100 mg of dry weight were used. The total lipid sample was then extracted with a mixture of 60% chloroform and 40% methanol (Merck, Darmstadt, Germany) at 100°C and 13.8 MPa.The crude extract was then concentrated under a stream of nitrogen and vacuum and heart total lipid (HTLip, expressed in g *per* g of dry heart muscle) were determined.Each sample was reconstituted in 20–30 volumes of ice-cold acetone to separate neutral lipids from polar lipids Because the proportion of neutral lipids obtained were negligible, only the lower phase corresponding to polar lipids was saponified in methanolic NaOH 0.5 N at 70°C for 20 min. Fatty acids were then prepared with boron-trifluoride-methanol (14 g BF_3_/L CH_3_OH, Merk-Schuchardt, Germany) in order to give fatty methyl esters (FAME) according to the procedure of [37].

FAME were analysed by liquid-gas chromatography in a Hewlett Packard HP 6890 Series GC System according the chromatographic conditions described in [36].

The presence of C13:0 fatty acid on samples was confirmed using a GC-MS Bruker Scion 456 equipped with a BR-Swax 30×0,25 µm column. Conditions were as follow: Inlet temperature −250°; Inlet mode - split 20 mL/min; He 1,2 mL/min column constant flow and oven temperature range from 120°C (for 5 min) to 240°C (for 10 min) with a ramping rate of 4°C/min; Ionization source: 70 eV electron ionization; and the GC-MS operated in full scan mode from 40–450 Da. To detect the subject compound ion extraction m/z 74 the m/z 87 were made.

The unsaturation index (UI), a measure of the number of double bonds within a sample, was calculated as the sum of the percentage of each unsaturated fatty acid multiplied by the number of double bonds within that fatty acid [38].

### Data analysis and interpretation: morphometric and meristic analysis

The statistical package SPSS for Windows (IBM, version 20.0) was used for data treatment and statistical analysis. Data transformations were used when appropriate.

The statistical analysis was applied following [14] which compared morphometric and meristic characters of *P. marinus* ammocoetes captured in several Portuguese river basins.

Briefly, analyses were performed separately for morphometric and meristic characters, and morphometric data were statistically adjusted to eliminate the influence of allometric growth as described in [14].

Outliers were detected by regression analysis of morphometric characters against total length, and by scatter plots of residual versus predicted values [39], resulting in the elimination of morphometric data for 19 lampreys (n = 18 from Group 1 and n = 1 from Group 3).

Of the 23 morphometric characters used in this analysis, 22 showed a linear relationship with total length (*P<*0.05). For character O, the only morphometric variable uncorrelated with total length (*P*>0.05), no size adjustment of the data was performed. For each of the 22 morphometric characters linearly related with total length, an analysis of covariance (ANCOVA) was employed to test for differences in allometric relationships among samples (i.e. geographical groups) and to estimate the common within-group regression slopes [39]. According to the ANCOVA analyses, within-group regression slopes were significantly different (df = 2, 201; *P<*0.05) for six of the morphometric characters (d-D_1_; H; d; hco; B_1_-B_7_; HW); and thus size adjustment was based on the common within-group slopes and was performed following a modification of the allometric formula given by [40], as described in [14].

A multivariate analysis of variance (MANOVA) was used to assess the main and interaction effects of categorical variables (gender and geographical groups) on the 23 dependent morphometric variables. Highly significant differences (*P*<0.001) were found between gender and groups, but not for the interaction effect (gender × group; *P*>0.05) (Table 1). Consequently, 10 morphometric variables were removed from further analysis to eliminate the influence of sexual dimorphism among morphometric characters (Table 2).

**Table 1:** MANOVA multivariate test with sex and geographical groups as factors, and adjusted morphometric characters as dependent variables.

Effect	Pillai's Trace value	*F*	df	*P*	Partial ε^2^	Obs. Power
Group	0.581	3.186	(46; 358)	<0.001	0.290	1.00
Sex	0.536	8.928	(23; 178)	<0.001	0.536	1.00
Group × Sex	0.245	1.086	(46; 358)	> 0.05	0.122	0.97

**Table 2:** Mean of adjusted morphometric characters used for the morphological analysis of *P. marinus*.

Morphometric	Male	Female	MANOVA (*F* statistic; df = 1)
d	3.978	3.945	9.246^***^
d-a	6.449	6.455	3.667*^NS^*
a-C	5.463	5.448	3.603*^NS^*
B_7_-C	6.541	6.545	12.366^**^
B_7_-a	6.133	6.145	12.195^**^
d-D_1_	6.099	6.104	0.057*^NS^*
d-eD_1_	6.313	6.316	0.000*^NS^*
d-D_2_	6.379	6.384	0.260*^NS^*
D_2_-C	5.643	5.630	1.970*^NS^*
lD_1_	4.671	4.670	0.096*^NS^*
lD_2_	5.385	5.369	0.484*^NS^*
D-D	3.682	3.710	2.452*^NS^*
H	3.995	4.068	50.131^***^
d-O	4.130	4.093	31.630^***^
O	2.185	2.176	0.107*^NS^*
O-B_1_	3.023	3.006	0.524*^NS^*
Hco	3.790	3.773	7.432^**^
d-B_1_	4.486	4.457	25.592^***^
B_1_-B_7_	4.470	4.466	0.631*^NS^*
d- B_7_	5.165	5.149	12.228^**^
d-n	4.010	3.972	18.027^***^
IO	3.857	3.832	15.328^***^
HW	4.000	3.990	1.912*^NS^*

Tests of Between-Subjects Effects from the MANOVA for the factor sex (presented in Table 1), are also presented.

Acronyms of variables as defined in Figure 2; *NS* P>0.05;* P<0.05;** P<0.01; *** P<0.001.

No significant relationship (*P<*0.05) was found between the meristic characters and total length and thus, no size adjustment was performed. Outliers were detected by the SPSS Boxplot procedure following [14], which resulted in the elimination of 13 specimens (n = 7 Group 1, n = 3 Group 2 and n = 3 Group 3).

A permutational multivariate analysis of variance (PERMANOVA, two-way crossed design) was used to assess the main and interaction effects of two factors (gender and geographical groups) on the meristic variables. No sexual dimorphism effect (*P*>0.05) nor geographical group (*P*>0.05) was found among the meristic characters (Table 3), and consequently meristic data was not subsequently analysed with a multiple discriminant analysis (MDA).

**Table 3:** PERMANOVA results for the two-way crossed design, with geographical group and sex as factors, and meristic characters as variables.

Source	Df	SS	MS	Pseudo-F	P(perm)	Unique perms
Group	2	0.821	0.410	1.498	0.193	9942
Sex	1	0.569	0.569	2.078	0.124	9951
Group × Sex	2	0.517	0.258	0.943	0.437	9955
Residual	181	49.592	0.274			
Total	186	51.493				

Morphometric data from the three groups defined *a priori* were compared separately by means of a MDA.

Since the groups formed *a priori* varied markedly in size, a stratified (river/gender) random sample from the larger group (i.e. Group 1) was performed to reduce their size to a level comparable with the smaller groups (i.e. Groups 2 and 3), following recommendations for sample sizes in MDA analysis [41]. Consequently, the MDA was run with a subsample of 36 individuals from Group 1, all individuals from Group 2 (n = 28) and from Group 3 (n = 23). The computational method used to derive the discriminant function was the stepwise method with the selection rule to maximize Mahalanobis *D* ^2^ between groups [41], and remaining MDA related procedures followed [14].

### Data analysis and interpretation: heart fatty acid profiles

The statistical package SPSS for Windows (IBM, version 20.0) was used for data treatment and statistical analysis. Data transformations were used when appropriate. Since the distribution of fatty acid percentages is binomial, an arcsine transformation of fatty acid data was used prior to statistical analysis to meet assumptions of normality, independence and homocedasticity.

The integrated chromatogram values for each fatty acid were expressed as a percentage of the total sum of fatty acids identified in order to eliminate concentration effects.

Multivariate analysis of variance (MANOVA) was used to see the main interaction effects of categorical variable (gender and geographical groups) on multiple interval variables (fatty acids) and to test our hypothesis that distinct geographical groups of sea lampreys present distinct heart phospholipids fatty acid composition. Significance of the MANOVA was evaluated with Wilk's lambda. Multiple discriminant analysis (MDA) was used to identify which fatty acid contributed most to the differences in heart tissue composition among geographical groups.

Once again, for the MDA a stratified (river/gender) random sample from the larger group (i.e. Group 1) was performed to reduce their size to a level comparable with the smaller groups (i.e. Groups 2 and 3). Consequently, the MDA was run with a subsample of 36 individuals from Group 1, all individuals from Group 2 (n = 19) and from Group 3 (n = 19). Also, the number of independent variables must not exceed the smallest group size [41], and consequently, the MDA was run with a subsample of 19 fatty acids instead of the 30 fatty acids previously identified in each sample group. So, one must select few fatty acids for use in a particular analysis, generally choosing fatty acids that are expected to vary based on biological functions or, if no *a priori* hypotheses exist, simply have the greatest abundance in the sample set. Because, in phosphoglycerides, the most common of the phospholipids that constitute animal cell membranes seldom contain significant amounts of saturated fatty acids other than 16:0, 18:0, and to a lesser extent 20:0 [42] so all fatty acids either with chain length smaller than 10 carbons, or with chain length greater than 22 carbons were excluded; in the pool of monounsaturated fatty acids the C14:1 and the only odd chain fatty acid were excluded and in the pool of polyunsaturated fatty acids the C18:3ω3 and C18:3ω6 were excluded since they were not detected in any of the groups.

The remaining procedures regarding the MDA with the fatty acids were similar to the analysis performed with the morphometric characters.

The Pearson correlation test was used to analyse the relationship between heart total mass (HTM) and heart total lipid content (HTLip) for lampreys of each geographical group.

## Results

### Sexual dimorphism

From the 224 adult lampreys captured, 109 were males and 115 were females. The total length (TL) and body total mass (TM) of the sampled lampreys ranged from 63.9 cm to 97.9 cm (mean TL = 86.4 cm) and from 770 g to 1806 g (mean *M*_T_ = 1188 g), respectively. Significant differences were found (ANCOVA; *F*_(1, 221)_ = 8.153, *P*<0.01) when comparing male (*y* = 0.0898*x* ^2.1209^; *r* ^2^ = 0.61; *d.f.* = 107;*P*<0.001) and female length-weight relationship (*y* = 0.0568*x* ^2.2342^; *r* ^2^ = 0.72; *d.f.* = 113;*P*<0.001). Generally, males tend to be longer while females are heavier, and differences tend to increase with length.

Gender related differences were found in 10 of the 23 morphometric characters analysed (Table 1; MANOVA, *P*<0.001). In general, males have larger cephalic regions including longer d, d-O, hco, d-B_1_, d-n and IO; while females have a tendency to longer and larger trunks (B_7_-a and H, respectively) (Table 2). No significant differences were found between genders for the analysed meristic characters (Table 3; PERMANOVA, *P*>0.05).

### Morphometric analysis

**Figure 3:**
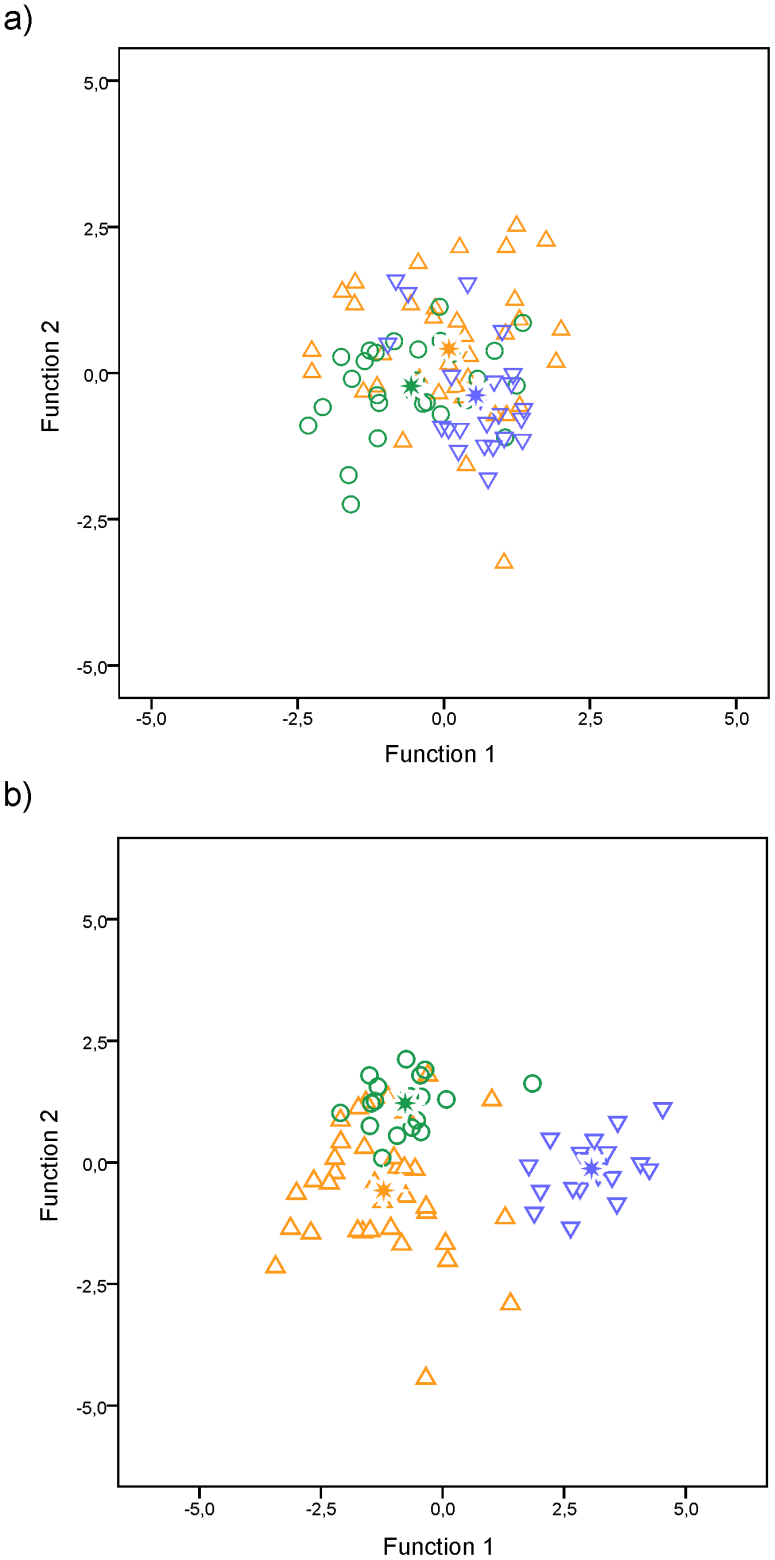
Plot of the discriminant *Z* scores and group centroids of discriminate functions 1 and 2 for the three groups of adult sea lampreys based on (a) morphometric characters and (b) fatty acid composition of heart total lipids. ▵ - Group 1: Minho, Lima, Cávado, Douro, Vouga and Mondego basins; ○ - Group 2: Tagus; ▿ - Group 3: Guadiana.

The regressions for Z scores from discriminant functions 1 and 2 of discriminant analysis against total length were not significant (*r* ^2^ = 0.011, df = 86, *P* = 0.340; and *r* ^2^ = 0.021, df = 86, *P* = 0.176), indicating that size effects had been removed from the adjusted morphometric variables. Discriminant functions are statistically significant (Wilk's lambda, *P*<0.001), and all pairs of groups showed statistically significant differences (df = 3, 82; *P*<0.05). The stepwise analysis revealed that three morphometric characters contributed significantly to the MDA (O, lD_2_, B_1_-B_7_). The Z scores and centroids from discriminant functions 1 and 2 were plotted against each other to develop a graphic representation of the relationship among groups (Fig. 3a). The two discriminant functions account for 59.3% and 40.7% of total variation. The total classification rate estimated from cross-validation procedure was 54%, ranging from 38.9 to 73.9% (Table 4). Press's Q test revealed that the classification accuracy is statistically significant better than chance (Press's Q = 16.759, df = 1, *P*<0.001).

**Table 4:** Classification results obtained with the stepwise discriminant analysis cross-validation for morphometric characters to determine the predictive accuracy level of the discriminant functions.

Groups	*N*	Percent correct	Predicted Group Membership (count)		
			Group 1	Group 2	Group 3
Group 1	36	38.9	14	11	11
Group 2	28	57.1	7	16	5
Group 3	23	73.9	5	1	17
Total	87	54.0			

*N*, number of individuals.

The interpretation of the plot in figure 3a indicates that Function 1 is the primary source of separation between Group 2 (river Tagus' lampreys) and Group 3 (river Guadiana's lampreys); whereas Function 2 discriminates Group 1 (Northern river basins' lampreys) from the remaining. Discriminant loadings and potency index were used to assess the contributions of the three discriminant morphometric variables (Table 5). High correlations between the discriminant loadings of the variable O with the first function, and lD_2_ and B_1_-B_7_ with the second function identified the variables with the best discriminatory power for each axis (Table 5). The character O was the variable with the highest potency index value and can be considered the most important morphometric character to distinguish adult *P. marinus* entering Tagus (mean O = 2.15) and Guadiana (mean O = 2.22) river basins (Table 5). Group 1 lampreys have intermediate size eyes (mean O = 2.18), longer second dorsal fins (mean 1D_2_ = 5.39) and shorter branchial lengths (mean B_1_-B_7_ = 4.46) when compared with lampreys entering rivers Tagus (1D_2_ = 5.37; B_1_-B_7_ = 4.48) and Guadiana (1D_2_ = 5.36; B_1_-B_7_ = 4.47).

**Table 5:** Summary of discriminant loadings and potency index for morphometric and meristic variables.

Variables	Discriminant loadings		Potency index
	Function 1	Function 2	
O	0.74^*^	−0.13	0.33
lD_2_	−0.03	0.81^*^	0.27
B_1_-B_7_	−0.32	−0.60^*^	0.21

Acronyms of variables as defined in Figure 2; * largest absolute correlation between each variable and any discriminant function.

### Meristic analysis

No significant differences were found between meristic characters of lampreys belonging to the *a priori* defined geographical groups (Table 3; PERMANOVA, *P*>0.05) and no significant interaction between gender and group was detected (Table 3; PERMANOVA, *P*>0.05). The AFteeth, LFteeth and PFteeth showed high variation between specimens, whereas the AFrows and LFrows did not show variation among individuals (Table 6).

**Table 6:** Summary statistics for the meristic characters analysed in the sub sample of 201 sea lamprey individuals included.

Meristic	Mean	SD	Min.	Max
Myo	73.1	1.6	68	78
TNteeth	148.6	8.0	128	170
AFteeth	39.5	3.8	29	49
LFteeth	62.4	2.9	54	69
PFteeth	37.2	2.7	31	46
TNrows	25.2	0.4	24	27
AFrows	7.0	0.0	7	7
LFrows	8.0	0.0	8	8
PFrows	9.2	0.4	8	11
IOcusps	7.5	0.7	6	10

Acronyms of variables as defined in Figure 2; *SD*, standard deviation; *Min*, minimum; *Max*, maximum.

### Tissue fatty acid profile

Individuals of Group 2 showed significantly higher values of heart total mass (HTM) (*P*<0.001) than the other two groups. However, no significant differences were detected for HTM/BGM ratio among individuals of the three groups (*P*>0.05; Table 7). The values of the HTM/BGM ratio were 0.25% for groups 1 and 2 and 0.24% for Group 3 (Table 7).

**Table 7:** Mean (± standard deviation) heart total mass (HTM, g), heart total mass/body gutted mass ratio (HTM/BGM expressed in percentage), mean heart total lipids (HTLip, expressed in g *per* g of dry tissue), heart water loss (H_Water_, expressed as percentage) and sex ratio of sea lamprey individuals analysed.

Variables	Group 1	Group 2	Group 3
HTM	2.89±0.5^*b^	3.34±0.4^*a,c^	2.76±0.3^*b^
HTM/BGM	0.25	0.25	0.24
HTLip	0.24^*b,c^	0.15^*a,c^	0.31^*a,b^
H_Water_	73.07±2.7	71.23±1.9	75.32±1.2
Sex Ratio (male/female)	0.48	0.46	0.68

Cases in which the relative amounts of a fatty acid are significantly different (*P*< 0.001) among the groups are marked with signs: *a: significantly different from Group 1; *b: significantly different from Group 2; *c significantly different from Group 3.

The heart total lipid content (HTLip) revealed significant differences (*P*<0.001) among the individuals of the three groups with sea lampreys of Group 2 showing the lowest value (15.3%), and the individuals of Group 3 presenting the highest value (30.8%) (Table 7). For each of the three groups, no significant correlation was found between HTM and HTLip.

The fatty acid profile of the heart tissue phospholipids varied among individuals of the three groups (Table 8). Gender had no significant effect (MANOVA, *P*>0.05) in fatty acid relative composition of phospholipids of heart tissue, whereas geographical groups exhibited a significant effect (MANOVA, *P*≤0.001). The interaction gender*geographical group had no significant effect in this variable (MANOVA, *P*>0.05).

**Table 8:** Relative amounts, as percentage of sum (mean ± sd), of fatty acids in heart tissue total lipids of sea lamprey individuals analysed.

	Fatty acid	Group 1	Group 2	Group 3
SFA	C10:0	2.84±2.09^*b^	0.74±1.10^*a;c^	3.36±1.54^*b^
	C12:0	0.08±0.16^*c^	0.06±0.05	ND
	C13:0	7.51±5.76^*b;c^	2.20±4.22^*a;c^	13.47±5.99^*a;b^
	C14:0	1.50±0.99^*c^	1.75±0.46^*c^	0.70±0.32^*a;b^
	C15:0	ND	ND	0.15±0.14^*a;b^
	C16:0	15.92±4.86^*b;c^	20.39±2.26^*a;c^	10.97±1.90^*a;b^
	C17:0	ND	ND	0.90±0.23^*a;b^
	C18:0	7.06±1.48^*b;c^	9.13±1.49^*a;c^	5.55±0.95^*a;b^
	C20:0	0.20±1.63^*b;c^	0.88±2.10^*a;c^	ND
	C22:0	0.22±0.62	0.28±0.30	ND
	ΣSFA	39.52	39.32	37.92
MUFA	C14:1	ND	0.06±0.06	ND
	C16:1	9.92±4.2^*b;c^	13.69±2.59^*a;c^	6.09±1.69^*a;b^
	C18:1ω9	16.64±6.23^*b^	22.95±2.67^*a;c^	12.86±2.55^*b^
	C20:1ω9	0.49±0.47^*b;c^	1.0±0.46^*a;c^	0.28±0.09^*a;b^
	C22:1ω9	ND	0.14±0.19	ND
	ΣMUFA	28.40	38.33	19.67
PUFA	C18:2ω6	0.29±0.23	0.35±0.14	0.30±0.21
	C20:2	0.14±1.20 ^*b;c^	0.32±3.11^*a;c^	ND
	C20:3ω6	ND	0.16±0.33	ND
	C20:3ω3	2. 69±1.48	3.10±1.07	3. 15±0.95
	C20:4ω6	ND	0.19±0.18	ND
	C20:5ω3	2.60±0.99	2.34±0.61^*c^	3.11±0.54^*b^
	C22:2ω6	0.12±0.52	ND	ND
	C22:5ω3	3.46±1.73	3.45±0.92	4.25±0.78
	C22:6ω3	8.21±4.62	6.32±1.74^*;c^	10.45±3.14^*b^
	ΣPUFA	18.83	17.72	21.38
	Σ(PUFA+MUFA)	47.23	56.05	41.05
	ΣUFA/ΣSFA	1.29	1.45	1.24
	Σω3	16.96	15.21	20.96
	Σω6	0.29	0.70	0.30
	C22:6ω3/C20:5ω3	3.16	2.70	3.36
	ΣEPA+DPA+DHA	14.27	12.11	17.81

SFA, saturated fatty acids; MUFA, monounsaturated fatty acids; PUFA, polyunsaturated fatty acids. Cases in which the relative amounts of a fatty acid are significantly different (*P*< 0.001) among the groups are marked with signs: *a: significantly different from group 1; *b: significantly different from group 2; *c significantly different from group 3. Fatty acids C6:0 and C8:0 are not presented because were not detected in each one of the three groups. ND, not detected.

The predominant class of fatty acids in heart tissue was the saturated fatty acids (SFA), with percentages that ranged from 37.92% in individuals from Group 3, to 39.52% in individuals of Group 1 (Tab. 8). The major exception was recorded in sea lampreys from Group 2, where the percentage of SFA was similar to that of monounsaturated fatty acids (MUFA). In what concerns MUFA, individuals of Group 3 were characterized by the lowest values (19.67%), and individuals of Group 2 were characterized by the highest values (38.33%). Polyunsaturated fatty acid (PUFA) relative percentages varied between 17.72% (Group 2) and 21.38% (Group 3) (Table 8).

The predominant fatty acids detected were C16:0, C18:0 and C13:0 for SFA; C18:1ω9 and C16:1 for MUFA; and C22:6ω3 (DHA), C22:5ω3 (DPA) and C20:5ω3 (EPA) for PUFA. The percentage content of EPA and DHA demonstrates the dominance of DHA over EPA (DHA/EPA ratio) Although occurring at relative low amounts, several odd chain fatty acids like C13:0; C15:0; C17:0 and C17:1, were also present in heart tissue fatty acid profiles (Table 8). The unsaturated-to-saturated ratio (UFA/SFA) was used as an indirect indicator of the membrane fluidity (Table 8), since it has been previously reported that membranes with high UFA/SFA ratio show a high fluidity [43]. Individuals from Group 2 presented the highest value (1.45), and individuals of Group 3 exhibited the lowest value (1.24), but no significant differences were observed (*P*>0.05). The unsaturation index (UI) was higher in Group 3 (132), followed by Group 1 (125) and by Group 2 (116), but no significant differences were observed (*P*>0.05).

The MDA for the 19 fatty acids proved to be statistically significant and the overall corrected classification rate estimated from cross-validation procedure was 83.8% (Fig. 3b). All lampreys from Group 3 were correctly classified (100%) and not mixing with the sea lampreys from any of the other groups, reflecting a typical heart fatty acid signature for individuals of this group (Fig. 3b, Table 9). It is also interesting to note that, 89.5% and 72.2% of the individuals from groups 2 and 1, respectively, were also correctly classified (Table 9). The fatty acids that contributed to the segregation of groups were the SFA C17:0 (35.9%) and C16:0 (25%); the MUFA C18:1ω9 (14.1%); and the PUFA C20:3ω6 (4.7%) and C22:2ω6 (3.3%). The fatty acid with the highest potency index that contributed for the separation of the sea lamprey groups was C17:0. Press's Q test revealed that the classification accuracy was significantly better than chance (Press's Q = 754.735 *gdl* = 1; *P*≤0.001).

**Table 9:** Classification results obtained with the stepwise MDA cross-validation for heart tissue fatty acids to determine the predictive accuracy level of the discriminant functions.

Number of individuals classified into group					
Groups	N	Percent Correct	North/Central	Tagus	Guadiana
North/Central	36	72.2	26	8	2
Tagus	19	89.5	1	17	1
Guadiana	19	100	0	0	19
Total	74	83.4	–	–	–

## Discussion

### Morphological variation and potential adaptation in the proposed groups

Sexual dimorphism among mature lampreys is well known and appears to be similar in all lamprey species [44]. This study data showed that male adult sea lampreys captured in the beginning of the spawning migration weight less than females, and although some additional subtle morphometric differences between genders were found, no obvious secondary sex characters were detected. Males show an increased prebranchial length and oral disc size, while females have a longer and wider trunk, similar to the findings of [45] for the Arctic lamprey *Lethenteron camtschaticum* (Tilesius, 1811). A larger head in males is most likely related with distinctive behaviours during the spawning period, in particular nest construction and agonistic behaviour when competing for females. A stronger suctorial capacity provided by larger oral discs may be an important characteristic to maximize reproductive success among males. Similar results were described for Southern Hemisphere species of the genera *Geotria* and *Mordacia*, of which an increase in size of the male oral disc, associated with an extension in the length of the preorbital region, was observed [46], [47]. The larger trunk observed in females is most likely related with the maximization of space for the development of the gonads, thus increasing fecundity. Elongated trunk in female lampreys was also described by [48] for the European river lamprey *Lampetra fluviatilis* L.

Morphologically, the classification rate estimated for animals from River Tagus (Group 2) was 57%, which means that most of the animals that entered this river to spawn presented similar characteristics, even though a considerable number of lampreys showed a morphological profile compatible with the other two groups. A poor discrimination rate (39%) was found for the northern lampreys pooled in Group 1 (i.e., rivers Minho, Lima, Cávado, Douro, Vouga and Mondego). This result indicates that specimens from this geographical region are morphologically more diverse when compared with sea lampreys entering Tagus and Guadiana basins. The wider range of abiotic scenarios like depth (see Fig. 1 for sea floor topography), observed in the oceanic area where northern lampreys occur, when compared with the other oceanic feeding areas discussed here, may lead to a higher morphological variability during the parasitic phase and, consequently, a lower classification rate of lampreys from Group 1. Also, this northern group may have lampreys originally from other oceanic regions, namely the Galicia Interior Basin or even the Biscay Abyssal Plain region, thus contributing to the low predicted group membership.

Three morphometric variables were considered significant in discriminating the three groups: eye length, second dorsal fin length and branchial length. The larger eye and longer branchial lengths of the Guadiana lampreys can be indicative of deeper feeding grounds, and the need for a more efficient mechanism of blood oxygenation. Comparatively, the relatively smaller eye and branchial length of the Northern lampreys may reveal shallower feeding grounds, and a less stressful demand for oxygen. Interestingly, the Northern lampreys with the lower classification rate, for both morphometric and heart fatty acid characters, was composed by individuals with longer second dorsal fins and thus, in theory, more capable for longer dispersions. The Guadiana lampreys had the highest classification rates among the three groups and the shortest second dorsal fin.

### Heart total mass/body gutted mass ratio in the proposed groups

The values of HTM/BGM ratio obtained for all individuals of the three geographical groups were similar (∼0.25%). This value is consistent with previous studies also with sea lamprey (∼0.3%; [49]), and similar to the characteristic values obtained for other poikilothermic vertebrates (0.08%–0.30% for fish, and 0.19% for amphibians and reptiles), but distant from the values usually determined in mammals (0.64%) [50].

In vertebrates this ratio reflects a direct relationship between the size of heart and oxygen consumption [51]. One would expect that lampreys from Group 3, with longer branchial lengths that may maximize oxygen uptake in less oxygenated habitats, would have a higher HTM/BGM ratio, but this was not the case.

Our results showed that individuals of Group 3 were characterized by the highest HTLip content. This result can be an indicator of increased rate of oxidative metabolism of fatty acids, since in cardiac muscle over 70% of energy consumed for electro-mechanical activity is covered by mitochondrial oxidation of fatty acids [52]. A study among 16 species of teleost fish revealed that glucose metabolism and fatty acid utilization increase with the increased energy demand [53]. According to this, our result suggests a higher demand for fatty acid oxidative metabolism of animals sampled in Group 3, comparatively to the individuals belonging to groups 1 and 2.

A lack of correlation between HTM and HTLip was expected because under physiological conditions, myocardial triacylglicerol stored in lipid droplets in the cytoplasm of the cardiac muscle cells is in a steady state condition, where no major alterations in the absolute amount of triacylglicerol fatty acids occur [52].

### Heart tissue fatty acid profile in the proposed groups

For all three groups, heart tissue fatty acid profile showed that SFA were the most representative. In groups 1 and 2 these were followed by MUFA and then PUFA whereas in Group 3, the MUFA and PUFA order was inverted. Moreover, since cardiac skeletal muscle contains a significant content of phospholipids, C16:0, C18:0, C16:1 and C18:1ω9 high relative amounts are in accordance with the fact that those fatty acids are the most common in the *sn*-1 position of phospholipids [38], [54]. Omega-3 fatty acids were also present In fact, the stable genetic basis of the fatty acid composition of heart tissue phospholipids [18], [20], [24], [25] in addition to the clear tendency for certain types of fatty acids to be incorporated into the *sn*-1 and *sn*-2 positions of the structural phospholipids, restrict the ability of the acyl chains to reflect diet.

Based on that, the fatty acid pool of polar lipids is considered stable over time and studies done with two stocks of reared Atlantic cod had already demonstrated it [18].The C13:0 showed higher values than expected and the explanation for these results is not known. However, this fatty acid could be associated to the presence of microbial sources considering some authors that C13:0 could be a useful of microbial presence on detritus [55]–[58].

Although ectothermic animals appear to increase the membrane content of unsaturated fatty acids in response to colder temperatures, a clear and direct relationship between specific unsaturated fatty acids and quantitative measurements of membrane fluidity has not been demonstrated [54]. In fact, a given overall fluidity level can be met by various fatty acids compositions, and often the fluidity of cellular membranes can be adjusted by converting SFA to MUFA, while the PUFA levels remain unaltered [59]. Because our results revealed that the unsaturated/saturated fatty acid ratio and unsaturation index were not significantly different among groups, this could mean that different fatty acid signatures were not caused by a direct effect of temperature on the phospholipids of heart tissue, but it is reasonable to believe that fatty acid profiles are phenotypic characters that must be correlated with differences in the abiotic factors that characterized different habitats and results from adaptation processes.

### Physiological adaptation to environmental conditions

Fish species inhabiting areas where environmental conditions are relatively stable and constant may develop a specialization of their membranes phospholipids that allow them to adapt to the environment where they live [60]. Habitats are different in many ways (e.g. temperature, salinity, pressure), so it is reasonable to hypothesize that environmental differences may lead to adjustments of the expression of several genes, resulting, for instance, in distinct heart tissue fatty acid signatures. Then, considering the heart tissue fatty acid profile as a phenotypic variation, the presence of different fatty acid signatures likely indicates limited mixing among groups and may offer a practical measure for stock discrimination [18], [23]. The differences in the heart tissue fatty acid profile of individuals of the geographical groups seem to result from the influence of environmental factors during the oceanic trophic phase of the lampreys' life cycle and to the geographical isolation promoted by seabed topography.

If this hypothesis is correct, it is possible that the oceanic phase of the sea lamprey life cycle, following the dispersion period during the juvenile trophic migration, is represented by a much less mobile adult stage restricting the mixture of lampreys from different geographical groups. This limited dispersal in marine environment was also highlighted in a recent work by Spice et al. [61] with Pacific lamprey (*Entosphenus tridentatus* Gairdner in Richardson 1836) and supported by Silva *et al*. [28] that suggest that at least a fraction of the sea lamprey population can reach adult size in approximately 14 months of hematophagous feeding. Moreover, part of this parasitic period can be spent feeding in rivers and estuaries before the trophic migration to the sea [62], so the marine stage might be even shorter than one year.

### Marine trophic phase and target host species

Dispersal is often density-dependent in a wide variety of *taxa* [63]. Due to population density, dispersal may relieve pressure for resources in an ecosystem, and competition for these resources may be a selection factor for dispersal mechanisms [64].

It is likely that the absence of large pelagic fish species (inexistence of salmonids and drastic reduction of shads *Alosa* sp.) in the southwest and south coasts of Portugal, induces sea lamprey juveniles undergoing their marine trophic phase to target benthic fish species [65]. On the other hand, the northern Portuguese river basins, which have a higher proportion of individuals in relation to Tagus and Guadiana groups [14], [66], can prey upon pelagic fish and thus experience a wider dispersion throughout the neighbouring marine areas and, consequently, river basins. Sea lampreys from the west coast of Portugal apparently present some clues that indicate the existence of two different trophic pathways, one typical of a top predator of a marine food web with a planktonic base, and the other including both planktonic and benthonic species [65]. Since benthonic fish are usually less vagrant in the adult stage when compared with the more mobile pelagic species [67], the migrations of adult sea lampreys between feeding areas (i.e. stocks) would be attenuated, and differences in the fatty acid profile of heart most likely arise under those circumstances.

### Juvenile dispersal, feeding areas and spawning migration

The three sea lamprey groups here identified are probably associated to the three isolated abyssal plains (and/or nearby continental slopes) off western Iberian Peninsula, and it is likely that they constitute three different stocks. Throughout the juveniles' trophic migration it is likely that some mixture between groups occurs, particularly between the northern (Group 1) and the Tagus (Group 2) stocks, which would be in agreement with the dispersal phase of some marine fish species during the juvenile stage in the same geographical area [68]. The lack of genetic differentiation between groups (results not shown, unpublished data) corroborates this scenario: since adults present significant levels of differentiation at the morphological and physiological levels, and there is genetic mixing between groups, the juvenile migration is most likely accompanied by dispersal among basins. During the spawning migration, lampreys seem to preferentially move north, probably attracted by the exceptional freshwater flow originated in northern river basins, particularly those north of river Douro, inclusive. In fact, eight animals sampled in the northern group presented characteristics of the Tagus' group.

The bulk of the juvenile lampreys from the isolated Guadiana river basin (Group 3) probably migrate to the feeding areas located at the Horshoe Abyssal Plain or nearby areas, which is located on the southern Iberian margin off western the Mediterranean Sea, and return to spawn in their river of origin. The impact of the Mediterranean Outflow Water (MOW) in the potential feeding area of animals entering the River Guadiana is particularly evident between 500 to 1400 m and shows higher temperatures and salinities than the North Atlantic Central Water (NACW) [69]. The unique conditions caused by the MOW influence may be responsible for the high distinct heart tissue fatty acid profile found in lampreys from group 3, as revealed by the 100% predictive accuracy level. In the oceanic zone over the continental slope, from December to February the dominant current (depth up to 1200 m) is oriented northward (Ana Teles-Machado and Álvaro Peliz, unpublished data). This may impel adult sea lampreys approaching the continent in the beginning of the spawning migration to the north with the prevailing current. Moreover, near the continental shelf, the dominant current is southward, and migrating sea lampreys may be once again oriented northward attracted by the odours transported from the northern rivers basins, which present higher ammocoete densities and river discharges than the Tagus or Guadiana river basins. This might explain why the North-Central group showed the occurrence of eight lampreys from the Tagus group and two from the Guadiana group.

In conclusion, the significant morphological and physiological differences found between groups are most likely the result from the influence of environmental factors to which lampreys may have been exposed during the oceanic trophic phase of the life cycle, rather than derived from a genetic basis. This would imply that the oceanic phase of the sea lamprey life cycle is composed by a dispersion period during the juvenile migration, followed by a much less mobile adult stage, which will restrict the mixture of adult lampreys from different geographical groups, segregated by seabed topography.

### Implications for conservation

The population structure put in evidence in this work have important implications in terms of management and conservation of *P. marinus* in Portugal, where it is considered threatened. Three stocks of this species are apparently present in Atlantic waters off country: the northern, the Tagus and the Guadiana stocks. The first includes individuals from Minho, Lima, Cávado, Douro, Vouga and Mondego river basins and, possibly, from North-western Spain (Galician rivers; not included in this study). A considerable number of lampreys still use the above referred basins for reproduction [66], except in River Douro, where apparently there are no suitable conditions for nest building in the available 20 km of river stretch downstream of the first obstacle [3]. The probable existence of a common stock in north-western Iberian waters reinforces the need for international joint efforts to manage this halieutic resource, commercially exploited both in Portuguese and Spanish watersheds.

Tagus and Guadiana stocks are, however, priority in conservation terms. The number of lampreys entering these basins, particularly in the southern Guadiana river basin, is very scarce. The existence of a lamprey stock composed mainly by sea lampreys originally from the Guadiana basin raises some concerns about the future of the species in its southern limit of distribution, mainly due to the hydric stress known to occur in this basin, and exacerbated by the potential effects of climate change.
